# Quinolone Resistance Mechanisms among Extended-Spectrum Beta-Lactamase (ESBL) Producing *Escherichia coli* Isolated from Rivers and Lakes in Switzerland

**DOI:** 10.1371/journal.pone.0095864

**Published:** 2014-04-22

**Authors:** Katrin Zurfluh, Helga Abgottspon, Herbert Hächler, Magdalena Nüesch-Inderbinen, Roger Stephan

**Affiliations:** Institute for Food Safety and Hygiene, Vetsuisse Faculty University of Zurich, Zurich, Switzerland; Aligarh Muslim University, India

## Abstract

Sixty extended-spectrum β-lactamase (ESBL)-producing *Escherichia coli* isolated from rivers and lakes in Switzerland were screened for individual strains additionally exhibiting a reduced quinolone susceptibility phenotype. Totally, 42 such isolates were found and further characterized for their molecular (fluoro)quinolone resistance mechanisms. PCR and sequence analysis were performed to identify chromosomal mutations in the quinolone resistance-determining regions (QRDR) of *gyrA*, *gyrB*, *parC* and *parE* and to describe the occurrence of the following plasmid-mediated quinolone resistance genes: *qepA*, *aac-6′-Ib-cr*, *qnrA*, *qnrB*, *qnrC*, *qnrD* and *qnrS*. The contribution of efflux pumps to the resistance phenotype of selected strains was further determined by the broth microdilution method in the presence and absence of the efflux pump inhibitor phe-arg-β-naphthylamide (PAβN). Almost all strains, except two isolates, showed at least one mutation in the QRDR of *gyrA.* Ten strains showed only one mutation in *gyrA*, whereas thirty isolates exhibited up to four mutations in the QRDR of *gyrA, parC* and/or *parE*. No mutations were detected in *gyrB.* Most frequently the amino-acid substitution Ser83→Leu was detected in GyrA followed by Asp87→Asn in GyrA, Ser80→Ile in ParC, Glu84→Val in ParC and Ser458→Ala in ParE. Plasmid-mediated quinolone resistance mechanisms were found in twenty isolates bearing QnrS1 (4/20), AAC-6′-Ib-cr (15/20) and QepA (1/20) determinants, respectively. No *qnrA, qnrB, qnrC and qnrD* were found. In the presence of PAβN, the MICs of nalidixic acid were decreased 4- to 32-fold. (Fluoro) quinolone resistance is due to various mechanisms frequently associated with ESBL-production in *E. coli* from surface waters in Switzerland.

## Introduction

Quinolones and fluoroquinolones (FQ) are broad-spectrum antimicrobial compounds and of critical importance in clinical use. They are the antimicrobials of choice in the treatment of acute gastrointestinal infections, urinary tract infections and respiratory tract infection. Resistance to FQ drugs increased in past years following the extensive and widespread use.

The mechanisms of action of (F)Qs is due to inhibition of DNA replication and resistance to (F)Qs is either based on (i) mutational alterations in the FQ target enzymes: DNA topoisomerase II (DNA gyrase) and topoisomerase IV, or (ii) plasmid-mediated quinolone resistance (PMQR) mechanisms or simply (iii) the decreased uptake of the drug due to the loss of a membrane-bound porin and/or drug extrusion via efflux pumps [1], [2]. PMQR mechanisms include target protection proteins of the *qnr* gene family, enzymatic modification gene *aac-(6′)-Ib-cr* and the efflux pump gene *qepA.* So far, five different *qnr* genes with a number of variations have been described: *qnrA, qnrB, qnrC, qnrD and qnrS* [3]–[7]. The Qnr proteins belong to the pentapeptide family of proteins and share common structural properties. AAC(6′)-Ib-cr differs from the aminoglycoside acetyltransferase, AAC(6′)-Ib, by two specific codon exchanges Trp102→Arg and Asp179→Tyr, which have been found to be necessary and sufficient for the ciprofloxacin (CIP) resistance phenotype [8]. The PMQR mechanisms confer only low level resistance to quinolones and thus, they were thought to enable the occurrence of chromosomal mutation which then lead to clinically relevant resistance levels [9].

In a previous study [10], 42 of 60 extended-spectrum β-lactamase-producing *Escherichia coli* strains isolated from rivers and lakes additionally displayed increased levels of nalidixic acid (NA) and/or CIP resistance.

The spread of *E. coli* co-expressing (fluoro)quinolone resistance along with extended-spectrum β-lactamases into the environment, especially into water bodies such as rivers, streams, wastewater effluents and lakes is worrisome. Rivers are considered to be of special importance as a reservoir of resistance genes since they are recipients of bacteria from different sources, e.g., wastewater plants, water of urban or industrial effluents, or agricultural activities [11].

The aim of this study was to identify and characterize quinolone and fluoroquinolone resistance mechanisms in higher generation cephalosporin resistant *E. coli* isolated from rivers and lakes in Switzerland.

## Materials and Methods

### Bacterial Isolates

Forty-two of 60 extended-spectrum β-lactamase-producing *Escherichia coli* strains isolated from rivers and lakes during a previous study and additionally displaying increased levels of nalidixic acid (NA) and/or CIP resistance were selected for this study. The protocol for collecting the water samples and isolating the strains as well as the species identification, antibiotic sensitivity testing and the characterization of the ESBL-types were described in the previous study [10].

### MIC Determination of Nalidixic Acid and Ciprofloxacin

MICs for NA and CIP of all 42 strains were determined using Etest strips (bioMérieux, Marcy l’Etoile, France). Of selected strains, MICs for NA (Sigma-Aldrich, Switzerland) were determined by the broth microdilution method according the Clinical and Laboratory Standards Institute (CLSI) [12], in the absence and presence of 40 µg/ml phe-arg-β-naphthylamide (PAβN) (Sigma-Aldrich, Switzerland). As recommended by the CLSI, the *E. coli* ATCC25922 was included as quality control strain.

### Identification of Quinolone Resistance Determinants and DNA Sequencing

DNA was extracted by a standard heat lysis protocol. Amplification of the quinolone resistance-determining regions (QRDRs) of the *gyrA*, *gyrB*, *parC* and *parE* genes and PCR based detection of the plasmid-mediated quinolone resistance (PMQR) markers (*qnrA*, *qnrB*, *qnrC*, *qnrD*, *qnrS*, *aac(6′)-Ib-cr* and *qepA*) was performed using primers listed in Table 1. Custom-sequencing was performed by Microsynth (Balgach, Switzerland) and the nucleotide and translated protein sequences were analyzed with CLC Main Workbench 6.6.1. For database searches the BLASTN and BLASTP programs of NCBI (http://www.ncbi.nlm.nih.gov/blast/) were used.

**Table 1:** Target genes, primers and PCR reaction conditions used for strain characterization.

Target	Primer	Sequence (5′-3′)	Annealing temperature (°C)	Product size (bp)	Reference
Detection primers					
*qnrA*	QnrAm-F	AGAGGATTTCTCACGCCAGG	54	516	[39]
	qnrA_R	GCCATACCTACGGCGATACC			[40]
*qnrB*	qnrB_F	GATCGTGAAAGCCAGAAAGG	54	476	[41]
	qnrB_R	ATGAGCAACGATGCCTGGTA			[41]
*qnrC*	qnrC-F	GGGTTGTACATTTATTGAATC	47	447	[6]
	qnrC-R	TCCACTTTACGAGGTTCT			[6]
*qnrD*	qnrD fw	CGAGATCAATTTACGGGGAATA	54	582	[7]
	qnrD rev	AACAAGCTGAAGCGCCTG			[7]
*qnrS*	QnrSm-F	GCAAGTTCATTGAACAGGGT	54	428	[39]
	QnrSm-R	TCTAAACCGTCGAGTTCGGCG			[39]
*aac(6′)-Ib-cr*	aac(6′)-Ib_For	TTGCGATGCTCTATGAGTGGCTA	55	482	[42]
	aac(6′)-Ib_Rev	CTCGAATGCCTGGCGTGTTT			[42]
*qepA*	QEPfor	TGGTCTACGCCATGGACCTCA	56	1137	[43]
	QEPrev	TGAATTCGGACACCGTCTCCG			[43]
Sequencing primers					
*gyrA*	gyrA WF	AAATCTGCCCGTGTCGTTGGT	55	344	[41]
	gyrA WR	GCCATACCTACGGCGATACC			[41]
*gyrB*	gyrB-F	GAAATGACC CGCCGTAAA	55	272	[44]
	gyrB-R	ACGACCGATACCACAGCC			[44]
*parC*	parC WF	CTGAATGCCAGCGCCAAATT	55	168	[41]
	parC WR	GCGAACGATTTCGGATCGTC			[41]
*parE*	parE-F	CTGAACTGCTGGCGGAGATG	60	483	[44]
	parE-R	GCGGTGGCAGTGCGACGTAA			[44]
*qnrS*	qnrS1_orf_f	GTTGTAATGTGTTGATGTAACAGG	52	825	This study
	qnrS1_orf_r	CCCTATGTCTATTATTGCAAGG			This study

### Transferability of the PMQR Determinants

Conjugation experiments were performed with the plasmid-free recipient strain *E. coli* HK225 strain (*Strep* ^r^, Rif^r^) [13] in order to assess the transferability of the plasmid-mediated quinolone resistance markers *qnrS1*, *aac-6′-Ib-cr* and *qepA*. Four *qnrS1* (OW1E2, OW54E2, OW60E1 and OW95E1), five *aac-6′-Ib-cr* (OW8C2, OW29E1; OW55C1, OW77E1 and OW89E1) and one *qepA* (OW54E1) positive isolates were selected at random as putative conjugation donors. Briefly, single colonies of the donor and recipient were inoculated in LB broth (Difco Laboratories) and grown overnight at 37°C. Subsequently, equal volumes of the donor and recipient cultures were mixed and incubated overnight at 37°C without shaking. Serial dilutions were then plated on LB agar (Difco Laboratories) selection plates supplemented with a combination of 600 µg/ml streptomycin (Sigma-Aldrich, Switzerland), 100 µg/ml rifampicin (Sigma-Aldrich, Switzerland) and either 10 µg/ml cefotaxime (Sigma-Aldrich, Switzerland) or 20 µg/ml tetracycline (Sigma-Aldrich, Switzerland). The colonies from the selection plates were examined by pulsed field gel electrophoresis (PFGE) to ensure acquisition of the plasmid. PCR was performed to (i) identify the resistance genes acquired by the transconjugants and (ii) for replicon typing the plasmids harboring PMQR genes co-transferred to the transconjugants.

### Replicon Typing

Plasmids were assigned to incompatibility groups on the basis of the presence of specific replicon sequences identified by PCR [14].

## Results

Of the total of 60 ESBL-producing *E. coli* isolates, 42 co-expressing (F)Q resistance were further characterized. Based on the Etest assay, two strains showed intermediate resistance to NA, nine were classified as resistant to NA, two were resistant to NA and showed intermediate resistance to CIP and 29 were resistant to NA and CIP.

In 40/42 (95.2%) of the investigated isolates, at least one point mutation leading to amino acid substitution in any of the quinolone resistance-determining regions (QRDR) of *gyrA, parC* and *parE* was detected (Table 2). No mutations were identified in *gyrB*. High-level resistance to NA was always attributed to a single mutation in *gyrA*, whereas strains resistant to both tested (F)Qs exhibited at least one point mutation in two different topoisomerase genes; most frequently in *gyrA* and *parC*. A total of ten strains showed only one point mutation, nine had the predominate substitution in GyrA Ser83→Leu and one a Ser83→Val exchange. One strain (OW55E1) showed one substitution in GyrA (Ser83→Leu) and one in ParC (Ser80→Arg). Six strains had the following set of substitutions: GyrA (Ser83→Leu and Asp87→Asn), and ParC (Ser80→Ile). OW86E2 showed the same substitutions in GyrA, but in ParC (Glu84→Val). Twenty-one strains showed four substitutions in at least two different proteins: fourteen had substitutions in GyrA (Ser83→Leu, Asp87→Asn) in ParC (Ser80→Ile) and in ParE (Ser458→ Ala), five strains in GyrA (Ser83→Leu, Asp87→Asn) in ParC (Ser80→Ile, Glu84→Val), OW54E1 in GyrA (Ser83→Leu, Asp87→Asn) in ParC (Ser80→Ile, Glu84→Gly) and OW60C2 in GyrA (Ser83→Leu, Asp87→Asn) in ParC (Ser80→IIe) and ParE (Glu460→Asp), respectively. Strain OW1C2 exhibited unique substitutions in GyrA: besides the frequently substituted position Ser83, here (Ser83→Tre), also positions Ile112→Val, Leu127→Met, Ala128→Ser and Lys154→Arg were affected. Additionally, substitutions in ParC (Ser80→Ile) and in ParE (Ser458→ Ala) were detected.

**Table 2:** Quinolone resistance, amino acid substitutions in the QRDR of GyrA, ParC and ParE proteins in terms of amino acid positions and PMQR determinants in extended-spectrum β-lactamase (ESBL) producing *Escherichia coli* isolated from rivers and lakes in Switzerland.

Isolate a)	Origin	blaESBL	NA MIC (mg/ml)	CIP MIC (mg/ml)	Substitutions in the QRDR			PMQR determinants			
					GyrA	ParC	ParE	qnrS1	aac(6′)-Ib-cr	qepA	Additional resistance profile
OW60E1	Reuss	CTX-M-27	12	0.5	−	−	−	+	−	−	AM, CF, CTX, TE, S
OW54E2	Aare	CTX-M-55	24	0.5	−	−	−	+	−	−	AM, CF, CTX, TE, SMZ
OW2E1	Eibach	CTX-M-15	256	0.19	S83L	−	−	−	−	−	AM, AMC, CF, CTX, GM, TE, TMP
OW64E2	Rhein	CTX-M-15	256	0.19	Ser83L	−	−	−	−	−	AM, CF, CTX, S, SMZ, TMP
OW65E1	Rhein	CTX-M-15	256	0.19	Ser83L	−	−	−	−	−	AM, AMC, CF, CTX, SMZ
OW67E1	Rhein	CTX-M-15	256	0.19	S83L	−	−	−	−	−	AM, CF, CTX, SMZ, TMP
OW77E2	Goldach	CTX-M-15	256	0.19	S83L	−	−	−	−	−	AM, CF, CTX, TE, S, SMZ, TMP
OW8E1	Marbach	CTX-M-3	256	0.25	S83V	−	−	−	−	−	AM, CF, CTX, SMZ, TMP
OW63E2	Limmat	CTX-M-15	256	0.25	Ser83L	−	−	−	−	−	AM, AMC, CF, CTX, TE, S, C, SMZ, TMP
OW95E1	Lorze	CTX-M-15	256	1.5	S83L	−	−	+	−	−	AM, CF, CTX, TE, S, SMZ, TMP
OW1E2	Birs	CTX-M-55	256	2	S83L	−	−	+	−	−	AM, CF, CTX, TE, S, C, SMZ TMP
OW18E1	Reuss	CTX-M-15	>256	0.25	S83L	−	−	−	−	−	AM, CF, CTX, TE, S, SMZ, TMP
OW55E1	Aare	SHV-12	>256	1	S83L	S80R	−	−	−	−	AM, AMC, CF, CTX, TE, S, C, K, SMZ, TMP
OW38E1	Emme	CTX-M-3	>256	8	S83L, D87N	S80I	−	−	−	−	AM, CF, CTX, TE, S, K, SMZ, TMP
OW48E1	Aabach	CTX-M-1	>256	12	S83L, D87N	S80I	−	−	−	−	AM, CF, CTX, GM, TE, S, C, K, SMZ, TMP
OW3E1	Birs	SHV-12	>256	24	S83L, D87N	S80I	−	−	−	−	AM, CF, CTX TE, S, C, SMZ, TMP
OW65E2	Rhein	CTX-M-1	>256	24	S83L, D87N	S80I	−	−	−	−	AM, CF, CTX, TE, SMZ
OW76E2	Thur	CTX-M-14	>256	24	S83L, D87N	S80I	−	−	−	−	AM, CF, CTX, GM, TE, S, SMZ, TMP
OW86E2	Rotbach	CTX-M-27	>256	>32	S83L, D87N	E84V	−	−	−	−	AM, CF, CTX, TE, S, SMZ, TMP
OW4E2	Ergolz	CTX-M-27	>256	>32	S83L, D87N	S80I, E84V		−	−	−	AM, CF, CTX, TE, S, SMZ
OW14E1	Rhein	CTX-M-14	>256	>32	S83L, D87N	S80I, E84V	−	−	−	−	AM, CF, CTX, SMZ
OW15E1	Rhein	CTX-M-27	>256	>32	S83L, D87N	S80I, E84V	−	−	−	−	AM, CF, CTX, SMZ, TMP
OW18E2	Reuss	CTX-M-15	>256	>32	S83L, D87N	S80I, E84V	−	−	−	−	AM, CF, CTX, TE, S, SMZ, TMP
OW54E1	Aare	CTX-M-55	>256	>32	S83L, D87N	S80I, E84G	−	−	-	+	AM, CF, CTX, TE, S, C, SMZ, TMP
OW77E1	Goldach	CTX-M-15	>256	>32	S83L, D87N	S80I, E84V	−	−	+	−	AM, CF, CTX, TE, K, SMZ, TMP
OW89E1	Reuss	CTX-M-15	>256	>32	S83L, D87N	S80I, E84V	−	−	+	−	AM, CF, CTX, GM, TE, K, SMZ, TMP
OW1C2	Birs	CTX-M-15	>256	>32	S83T, I112V, L127M, A128S, K154R	S80I	S458A	−	+	−	AM, AMC, CF, CTX, GM, K, SMZ, TMP
OW3C1	Birs	CTX-M-15	>256	>32	S83L, D87N	S80I	S458A	−	+	−	AM, AMC, CF, CTX, GM, K, SMZ
OW5E1	Ergolz	CTX-M-14	>256	>32	S83L, D87Y	S80I	S458A	−	−	−	AM, CF, CTX, GM, S, SMZ, TMP
OW8C2	Marbach	CTX-M-15	>256	>32	S83L, D87N	S80I	S458A	−	+	−	AM, AMC, CF, CTX, TE, K, SMZ, TMP
OW14E2	Rhein	CTX-M-15	>256	>32	S83L, D87N	S80I	S458A	−	+	−	AM, AMC, CF, CTX, TE, S, K, SMZ, TMP
OW15C1	Rhein	CTX-M-15	>256	>32	S83L, D87N	S80I	S458A	−	+	−	AM, AMC, CF, CTX, GM, TE, K, SMZ, TMP
OW16E2	Rhein	CTX-M-15	>256	>32	S83L, D87N	S80I	S458A	−	+	−	AM, AMC, CF, CTX, GM, TE, K, SMZ, TMP
OW18C1	Reuss	CTX-M-15	>256	>32	S83L, D87N	S80I	S458A	−	+	−	AM, AMC, CF, CTX, GM, TE, S, K, SMZ, TMP
OW29E1	Binzmühleweiher	CTX-M-15	>256	>32	S83L, D87N	S80I	S458A	−	+	−	AM, AMC, CF, CTX, GM, TE, C, K, SMZ, TMP
OW50E1	Aabach	CTX-M-15	>256	>32	S83L, D87N	S80I	S458A	−	−	−	AM, CF, CTX, TE, S, C, SMZ, TMP
OW55C1	Aare	CTX-M-15	>256	>32	S83L, D87N	S80I	S458A	−	+	−	AM, AMC CF, CTX, GM, TE, K, SMZ
OW60C2	Reuss	CTX-M-79	>256	>32	S83L, D87N	S80I	E406A	−	−	−	AM, CF, CTX, TE, S, C, K, SMZ
OW60E2	Reuss	CTX-M-15	>256	>32	S83L, D87N	S80I	S458A	−	+	−	AM, AMC, CF, CTX, GM, TE, K, SMZ, TMP
OW77C1	Goldach	CTX-M-15	>256	>32	S83L, D87N	S80I	S458A	−	+	−	AM, CF, CTX, GM, C, K, SMZ, TMP
OW78E1	Rotbach	CTX-M-15	>256	>32	S83L, D87N	S80I	S458A	−	+	−	AM, AMC, CF, CTX, GM, TE, S, K, SMZ, TMP
OW80E1	Katzensee	CTX-M-15	>256	>32	S83L, D87N	S80I	S458A	−	+	−	AM, CF, CTX, GM, TE, C, K, SMZ, TMP

a ) see Zurfluh et al. [10] for comparison.

AM, ampicillin; AMC, amoxicillin-clavulanic acid; CF, cephalothin; CTX, cefotaxime; CIP, ciprofloxacin; GM, gentamicin; TE, tetracycline; S, streptomycin; C, chloramphenicol; K, kanamycin; NA, nalidixic acid; SMZ, sulfamethoxazole; TMP, trimethoprim.

QRDR: quinolone resistance-determining regions; PMQR: plasmid-mediated quinolone resistance mechanisms.

Twenty isolates (48%) harbored plasmid-mediated quinolone resistance (PMQR) genes. Most frequently the *aac-6′-Ib-cr* gene 15/42 (36%) was found, followed by *qnrS1* 4/42 (10%) and *qepA* 1/42 (2%). The *qnrA, qnrB, qnrC and qnrD* genes were not detected in this study.

Intermediate resistance to NA was associated either with the presence of the plasmid-mediated resistance protein QnrS1 since in two strains (OW54E2 and OW60E1) chromosomal mutations and other PMQR determinants were absent, or with the presence of general efflux pumps.

Conjugation experiments revealed that two of the four *qnrS1*, three of five selected *aac-6′-Ib-cr* but not the *qepA* bearing plasmid were transferable to the recipient strain *E. coli* HK225.

Plasmid incompatibility typing of the transconjugants revealed that both the *qnrS1* and *aac-6′-Ib-cr* genes were present on IncF plasmids. Furthermore, all transferred PMQR determinants were detected on the same plasmids as the *bla*_ESBL_ genes. The contribution to FQ resistance of the plasmids bearing the PMQR determinates was measured by the difference in the MICs (NA and CIP) of the recipient strain (*E. coli* HK225) compared to the transconjugants. The effect on the resistance to NA was minimal since the MICs increased only 1-3-fold. However, the resistance to CIP increased by factors of 4 to 16 in the presence of PMQR determinant harboring plasmids (Table 3).

**Table 3:** Plasmid-mediated quinolone resistance mechanisms, replicon types of transferred plasmids and quinolone resistance levels of the transconjugants of selected *E. coli* isolates.

Strain	Transferred gene	Replicon types	Etest MIC (µg/mL) [Fold-MIC increase vs. donor]	
			NA	CIP
*E. coli* HK225 Strep^R^Rif^R^		–	4	0.023
TC_OW54E2	*qnrS1*	F	12 [3]	0.38 [16]
TC_ OW95E1	*qnrS1*	F	8 [2]	0.25 [10]
TC_OW8C2	*aac-6′-Ib-cr*	F	4 [1]	0.094 [4]
TC_OW29E1	*aac-6′-Ib-cr*	F	6 [1.5]	0.094 [4]
TC_OW77E1	*aac-6′-Ib-cr*	F	4 [1]	0.094 [4]

*E. coli* HK225 Strep^R^Rif^R^, recipient strain resistant to streptomycin and rifamycin.

TC_OW54E2, transconjugant receiving the plasmid from the donor strain OW54E2.

In the presence of the efflux pump inhibitor PAβN, the MICs of NA decreased 4 to 32-fold. This finding confirmed that also efflux pump activity is involved in quinolone resistance (Table 4).

**Table 4:** MIC values of nalidixic acid in the presence and absence of Phe-Arg-β-naphthylamide in selected *E. coli* isolates.

	MIC Values (µg/mL)				Number of amino acid-relevant mutations		
Isolate	NA	NA +PAβN	Fold-decrease	PMQR genes	*gyrA*	*parC*	*parE*
OW1E2	128	16	8	*qnrS1*	1	*–*	*–*
OW1C2	>1024	64	>16	*aac–6′-Ib-cr*	5	1	1
OW3E1	>1024	128	>8	–	2	1	–
OW8E1	512	16	32	–	1	–	–
OW14E2	>1024	256	>4	*aac-6′-Ib-cr*	2	2	–
OW54E1	>1024	256	>4	*qepA*	2	2	–
OW54E2	32	2	16	*qnrS1*	–	–	–
OW55C1	>1024	64	>16	*aac-6′-Ib-cr*	2	1	1
OW55E1	>1024	128	>8	–	1	1	–
OW60E1	16	2	8	*qnrS1*	–	–	–
OW64E2	256	16	16	–	1	–	–
OW65E2	>1024	128	>8	–	2	1	–
OW77E1	>1024	128	>8	*aac-6′-Ib-cr*	2	2	–
OW86E2	>1024	128	>8	–	2	1	–
OW95E1	256	16	16	*qnrS1*	2	2	–
ATCC25922	<1	<1	1	–	–	–	–

## Discussion

So far, there are only few studies that have investigated the occurrence of (fluoro)quinolone resistant *Enterobacteriaceae* in surface waters [15]–[18]. Rivers and lakes are known to be a relevant putative reservoir of multiresistant bacteria since they collect surface waters containing material from different origins, e.g., wastewater plants, water from urban or industrial effluents, agricultural activities, and they harbor an immense antibiotic resistome, distributed to pathogenic and nonpathogenic bacteria [19].

FQ resistance in *E. coli* is associated predominantly with DNA topoisomerase II (DNA gyrase) and topoisomerase IV involving specific mutations in the QRDRs of *gyrA* and *parC* genes [2]. A single mutation in the QRDR of *gyrA* seems to be sufficient to generate a high-level quinolone, and a double mutation even a high-level fluoroquinolone resistance phenotype [20]. It is supposed that these mutations occur in a stepwise manner [2]. The effect of these mutations may alter the formation of hydrogen bonds, and the negative charge of the amino acids seem to be important for quinolone interactions with the DNA-gyrase-DNA complex [21].

As in previous studies, we mainly detected the substitutions in terms of amino acid positions GyrA Ser83→Leu and GyrA Asp87→Asn, which are located within the QRDR. Interestingly, OW1C2, besides harboring a substitution at position GyrA Ser83→Val, contained additional substitutions at position 112, 127, 128 and 154. So far, these four substitutions have been found in a *Raoultella planticola* and a *Klebsiella oxytoca* isolate (except for position 154), as shown by sequence alignments using the NCBI BLASTP program (accession-numbers: BAN58234.1 and CAQ76691.1). Hitherto, very few studies have focused upon the role of amino acid substitutions outside the QRDR [22], hence little is known about their effect on resistance levels. Our finding suggests that in addition to the standard QRDR, additional sites in GyrA seem to play a role in FQ resistance. Further studies thus need to be conducted in order to further characterize the role of rare substituions in GyrA.

In clinical *E. coli* isolates, quinolone resistance is mainly associated with mutations in *gyrA* whereas mutations in the topoisomerase IV genes, *parC* and *parE*, play a secondary role [2]. To date, mutations within *parC* and *parE* are always linked to mutations in *gyrA,* and it is supposed that mutations within these genes only occur if the strain already posses a reduced sensitivity to fluoroquinolones by mutations in DNA gyrase. Therefore, additional mutations in *parC* and *parE* are supposed to play an important role in the formation of highly resistant strains [2], [23]. As many as 71% of the isolates described in this study, in addition to showing one to two substitutions in GyrA, harbored substitutions in ParC. Interestingly, the substitutions in ParC were found predominantly at position Ser80, which, apart from substitutions in GyrA, is one of the most common alterations in FQ-resistant clinical isolates [24]. This finding is suggestive of the anthropogenic origin of these strains that were isolated from the aquatic environment.

PMQR is a phenomenon of increasing importance. The most relevant PMQR genes known to date are the acetyltransferase gene *aac(*6′)-Ib-cr, the *qnr*-like genes and genes encoding efflux pumps, such as *qep*A [25], [26].

In the isolates described in this study, PMQR determinant *aac(6′)-Ib-cr* was the most prevalent (36%), followed by *qnrS1* (10%) and *qepA* (2%). The relatively high prevalence of *aac(6′)-Ib-cr* is not surprising since several reports showed that this gene is often present on the same plasmid as *bla*_ESBL_ [27], [28]. QnrS-producing strains were frequently described in aquatic environments, and - as shown by a study by Cattoir and Co-workers [29] - *Vibrio splendidus,* an aquatic Gram-negative bacteria, was identified as a possible source for *qnrS-*like genes. Interestingly, in a previous study by Picão et al. [30], a *qnrS2* bearing *Aeromonas allosaccharophila* strain was isolated from Lake Lugano in the southern part of Switzerland. QnrS is found very rarely and only sporadically in clinical isolates in Europe [31]. Further, Liassine et al. [32] investigated human ESBL-producing *Enterobacteriacae* isolates for *qnrA, qnrB and qnrS* collected by a Swiss clinical microbiology laboratory. However, from 155 analyzed isolates, they found only *qnrB4* (3 *Citrobacter freundii,* 1 *Klebsiella pneumonia* and 1 *E. coli*) and *qnrB2* (1 *E. coli*). In contrast to our data, no *qnrS* variants were found in that study, indicating that *qnrS* is not characteristic for FQ-resistant human isolates in Switzerland.

Our conjugation experiments revealed that *qnrS1*, and *aac-6′-Ib-cr* harboring plasmids were transferable to FQ-susceptible strains. The resistance phenotypes of the resulting transconjugants confirmed previous observations that the contribution of PMQR determinants to fluoroquinolone resistance levels of the plasmid-bearing strains is minor compared with the chromosomal mutations in DNA gyrase. The proposed role of these genes is that they enhance resistance to fluoroquinolones, supplementing chromosomal resistance genes [33]. Poor transfer of *qnrS*-encoding plasmids is confirmative of a previous study describing a *qnrS*-harboring clinical *E. coli* isolate [31]. Nevertheless, our results demonstrate that *qnrS* determinants are indeed transferable and capable of conferring very low level resistance, also in the absence of chromosomally-encoded (F)Q-resistance determinants.

Replicon typing of the transconjugants revealed that both *qnrS1* and *aac-6′-Ib-cr* are present on IncF plasmids. Additionally, the genes coding for ESBL were detected on the same plasmids. CTX-M-15, the most prevalent CTX-M-type isolated from water bodies in Switzerland, is often associated with *aac(6′)-Ib-cr* resistance genes and has been located frequently on IncF group plasmids [34]. IncF type plasmids carrying CTX-M-15 are an inhomogeneous group of plasmids with sizes that vary between 50–200 kb and often carry more than one replicon (e.g., repFII, repFIA and/or repFIB) [34]. This multi-replicon plasmid-genotype was also observed in our study.

Besides chromosomal mutations and PMQR determinants, efflux pumps can also contribute in a more unspecific way to the (F)Q resistance phenotype. The effect of members of the resistance-nodulation-division (RND) family of membrane transporters can be investigated by inhibiting them with PAβN [35], [36]. In this study, the MICs of NA decreased 4 to 32-fold in the presence of the inhibitor, which clearly confirmed that these efflux pumps play an important role in quinolone resistance. Sáenz et al. [36] could already show that a single amino acid exchange in GyrA was not enough to confer a high level NA resistance phenotype in *E. coli.*

Recently, Sato et al. [37] investigated an *E. coli* isolate whose MICs of CIP, levofloxacin and norfloxacin exceeded the respective CLSI breakpoints, although it had no mutations in the QRDRs of either DNA gyrase or topoisomerase. The conclusion of their study was that FQ resistance can also arise due to concomitant acquisition of several PMQR determinates (in their study: QnrS1 and OqxAB, in combination with overexpression of acrAB-TolC and other chromosome-encoded efflux pumps). Our study yielded no isolate of this kind.

In summary, the acquisition of a high-level (F)Q resistance phenotype often includes combination of multiple mechanisms such as acquisition of mutations in DNA gyrase and topoisomerase genes as well as enhanced efflux and these processes can be accelerated by the presence of PMQR-determinants [2]. Our findings suggest that this holds true also for our FQ-resistant strains isolated from the aquatic environment.

It is possible that as a consequence of excretion of unmetabolized quinolones into wastewater by humans and animals, the environmental quinolone accumulation has contributed to the high prevalence of quinolone resistant *E. coli* in the water environment [9], [38].

This study provides further evidence for the importance of the water-related environment as a reservoir for this emerging threat of FQ resistance. Moreover, the resistance characteristics of the isolates described in this study indicate that multilateral exchange of genetic material between bacteria of both anthropogenic and environmental origins is currently occurring and presents a phenomenon of growing importance. It is, finally, worrisome that more than two thirds of ESBL producers carry resistance determinants against (F)Qs, thus defying the two currently most popular antibiotic options for human therapy, β-lactams and FQs.
